# Cardiovascular Disorders in the Context of Non-Alcoholic Fatty Liver Disease: A Literature Review

**Published:** 2014-01-12

**Authors:** Reza Karbasi-Afshar, Amin Saburi, Hossein Khedmat

**Affiliations:** 1 *Atherosclerosis Research Center, Baqiyatallah University of Medical Sciences, Tehran, Iran.*; 2 *Department of Cardiology, Baqiyatallah University of Medical Sciences, Tehran, Iran.*; 3 *Chemical Injuries Research Center, Baqiyatallah University of Medical Sciences, Tehran, Iran.*; 4 *Baqiyatallah Research Center for Gastroenterology and Liver Disease, Tehran, Iran.*

**Keywords:** *Heart* • *Coronary artery disease* • *Non-alcoholic fatty liver disease* • *Review*

## Abstract

Non-alcoholic fatty liver disease (NAFLD) is the leading cause of chronic liver disease in the United States and other industrialized countries, and the reported prevalence in the developing countries is also rather high. This disease is associated with a high rate of morbidity and mortality and damage to the other organs. The cardiovascular system is, perhaps, the most vulnerable organ to NAFLD adverse effects to the extent that most mortality associated with this disease is reportedly from the cardiovascular system rather than from the liver itself. In this article, we review the significant aspects of cardiovascular disorders associated with NAFLD, including the epidemiology of cardiovascular diseases in NAFLD patients, factors that interfere in this relationship like hypertension, severity of NAFLD, and age of the patients, and finally preventive strategies whose employment could significantly improve the outcome.

## Introduction

Non-alcoholic fatty liver disease (NAFLD) is the leading cause of chronic liver disease in the industrialized countries. Evidence suggests that it is probably as common in the developing world as it is in the developed world, with a prevalence up to 45% in the general population.^1^^, ^^2^ NAFLD is a term used to define a large spectrum of conditions, ranging from simple non-symptomatic steatosis to non-alcoholic steatohepatitis, and cirrhosis.^3^ Nevertheless, hepatic disorders are not the sole morbidity associated with NAFLD, and several extra-hepatic manifestations of this disease, including malignancies and cardiovascular events, threaten patients’ lives.^4^^, ^^5^

Cardiovascular disorders constitute major health threats in NAFLD patients. An increased rate of cardiovascular events has been reported in this population,^6^ while cardiovascular mortality in these patients has independently increased in those with a prominent metabolic syndrome.^4^ Moreover, patients with non-alcoholic steatohepatitis (NASH), a more destructive form of NAFLD, are probably at higher risk of atherosclerosis formation than patients with simple steatosis.^7^

In this article, we review the current literature on the existing data on potential associations between cardiovascular disorders and NAFLD. The research focuses on most recent articles (especially since 2010) and/or strongest data (prospective and large studies).

***Epidemiology of Coronary Atherosclerosis and Non-Alcoholic Fatty Liver Disease Associations***

Overwhelming data indicate powerful associations between NAFLD and the risk factors of cardiovascular disorders.^8^^-^^11^ Compared to the general population with confirmed non-steatotic liver, NAFLD patients have impaired flow-mediated vasodilatation, which is a reliable marker of subclinical atherosclerosis, and this impairment is independent of other established risk factors, including obesity.^12^ In a study of 312 consecutive patients undergoing elective coronary angiography, 34 patients with NAFLD diagnosis represented significantly decreased circulating bone marrow-derived endothelial progenitor cells (cEPCs) levels (all p values < 0.05), attenuated EPCs functions, and enhanced systemic inflammation compared to the controls. Multivariate analysis showed that the cEPC level [CD34 + KDR + (cells/105 events)] was an independent reverse predictor of NAFLD (OR: 0.78, 95%CI: 0.69–0.89; p value < 0.001).^13^

Moreover, several studies have shown that NAFLD can result in clinical atherosclerosis as well. In a study of 10153 Korean people participating in an occupational cohort, having fatty liver determined by ultrasonographic evaluation was associated with coronary artery calcification defined by computed tomography, and this association was independent from the conventional risk factors.^13^ In another interesting Korean study, 4023 subjects of no known history of either liver or coronary disease were evaluated with the same methodology of the previous one and the authors found that NAFLD was independently associated with coronary artery calcification, and interestingly a higher score of coronary calcification was associated with a higher prevalence of NAFLD.^14^ Almost similar to this result, in a study evaluating patients consecutively referred for elective coronary angiography, NAFLD was independently associated with more severe coronary artery disease.^15^ A community-based cohort of 2088 male Taiwanese workers showed that the diagnosis of NAFLD by ultrasonography was independently associated with the presence of ischemic changes in electrocardiography.^16^ Another study from Taiwan through multivariate analysis showed that the prevalence of NAFLD in asymptomatic patients increased with the severity of the coronary artery calcification score (≤ 100, 38.1%; 101-400, 58.3%; > 400, 64.3%; p value = 0.03).^17^ Yilmaz et al.,^18^ in their study of coronary flow reserve (CFR) in NAFLD patients found that over 42% of the patients had impaired CFR and this impairment was lowest in patients with more advanced liver fibrosis.

***Epidemiology of Coronary Artery Events among Non-Alcoholic Fatty Liver Disease Patients***

Several prospective studies have been conducted to examine whether NAFLD and cardiovascular disorders are somehow connected together. Hamaguchi et al.^19^ conducted a 5-year prospective cohort study of apparently healthy Japanese people who had come for check-up evaluations and studied them for the availability of NAFLD and the metabolic syndrome. At the end of the follow-up time, the authors found that the incidence of cardiovascular disorders in the NAFLD group was significantly higher than that in the non-NAFLD controls (2.2% vs. 0.3%, respectively; OR: 4.12; 95%CI: 1.58-10.75; p value = 0.004). Another study by the same authors confirmed the previous results; the authors expanded the study with a longer follow-up period (6.5 years) and found a similar relationship between death and cardiovascular disease.^20^ Wong et al.^21^ showed that NAFLD was an independent predictor of cardiovascular events (adjusted OR: 2.31; 95%CI: 1.46 to 3.64). A more recent prospective cohort study by Lazo et al.^22^ on 11371 adults followed for up to 18 years revealed that, compared to the participants without steatosis, those with NAFLD but normal liver enzyme levels had multivariate adjusted hazard ratios (HR) for deaths from cardiovascular disease of 0.86 (0.67 to 1.12). Similar results were obtained when the data were reanalyzed for the NAFLD patients with abnormal liver enzyme levels (OR: 0.59, 95%CI: 0.29-1.20). There are some prominent retrospective cohorts of very long follow-up periods,^23^^-^^28^ whose data are summarized in Table 1.

**Table 1:** Major retrospective cohort studies on the associations between non-alcoholic fatty liver disease (NAFLD) and cardiovascular events

Study authors	Follow-up (y)	Main results	NAFLD population size
Rafiq et al.^23^	18.5	The most common causes of death were coronary artery disease, malignancy, and liver-related death; with no difference between the NAFLD subtypes.	173 biopsy-proven NAFLD
Ekstedt et al. ^24^	13.7	Risk of death from cardiovascular disease was higher by a factor of 2 among 129 patients with non-alcoholic steatohepatitis than in the general population.	129 biopsy-proven NAFLD
Söderberg et al.^25^	28.0	Cardiovascular reasons were the main cause of death in the NAFLD population (30%). Non-alcoholic steatohepatitis (NASH) was associated with increased mortality from all causes and from cardiovascular disease.	118 biopsy-proven NAFLD (20% NASH)
Adams et al. ^26^	7.6	There were higher rates of cardiovascular mortality in NASH patients than in the general population.	420 biopsy/imaging diagnosis of NAFLD
Dam-Larsen et al. ^27^	20.4	There were higher rates of cardiovascular death in NAFLD patients. Mortality was not associated with histological grading.	170 biopsy-proven NAFLD
Wang et al.^28^	10.0	Risk of cardiovascular disease increased with increasing fatty liver status in both genders. The difference was not only present between individuals with fatty liver vs. non-fatty liver but also between the mild fatty liver and significant fatty liver groups. The odds ratio for every increment of fatty liver severity was 2.3 in the women (95%CI: 1.4-3.5) and 2.7 in the men (95%CI: 1.7-4.1).	462 imaging-diagnosed NAFLD

***Non-Alcoholic Fatty Liver Disease*** ***in the Presence of Metabolic Disorders***

Metabolic factors are the cornerstone of the pathogenesis of NAFLD,^8^ and it is supposed that most ominous events associated with NAFLD are due to the metabolic disorders in this disease. Diabetes mellitus is the main metabolic disorder and can lead to NAFLD or affect its prognosis. In type 2 diabetic patients with known coronary artery disease, Lautamäki et al.^29^ found that NAFLD diagnosed by magnetic resonance spectroscopy was independently associated with reduced myocardial perfusion. Targher et al.^30^ carried out a 5-year prospective nested case-control study in 2103 type 2 diabetic patients with a diagnosis of cardiovascular disorders at baseline. After adjustment for demographic data as well as conventional risk factors, the presence of NAFLD was significantly associated with an increased risk of cardiovascular events (OR: 1.84, 95%CI: 1.4-2.1; p value < 0.001). In another study by the same authors, involving around 3000 patients with type 2 diabetes, the prevalence of coronary artery disease was independently higher among patients with NAFLD than among those without this disease.^20^ Similar findings were also reported by another study on type 1 diabetes.^31^ In type 2 diabetic patients with well-controlled metabolic status, even mild elevation in liver enzymes was proved to be independently related to decreased insulin sensitivity and impaired brachial artery flow-mediated vasodilation.^32^ In children, NAFLD in overweight individuals has been proven to be associated with multiple cardiovascular risk factors, including higher serum levels of fasting glucose, insulin, total cholesterol, low-density lipoprotein cholesterol, and triglycerides as well as higher systolic and diastolic blood pressure and lower high-density lipoprotein cholesterol, than overweight and obese children without NAFLD (controls).^33^ Multivariable analysis in the same study revealed that the children with the metabolic syndrome had 5.0 (95%CI: 2.6-9.7) times the odds of having NAFLD as the controls. 

Gamma-glutamyltransferase (g-GT) is a subclinical indicator of insulin resistance,^34^ and it has been suggested that its high serum values represent early evidence for oxidative stress. Moreover, hepatic steatosis is characterized by an elevated level of g-GT,^35^ which is also a predictor of coronary artery disease.^36^ A large population-based study conducted in Germany reported that elevated gamma-glutamyl transpeptidase (GGT) levels in men with hepatic steatosis were strongly associated with higher mortality rates (HR: 1.98; 95%CI: 1.21-3.27).^35^ In the same study, when death due to cardiovascular reasons was evaluated, a more than twofold increased risk of cardiovascular disease mortality from elevated GGT levels for both sexes in an age-adjusted model was observed [HR: 2.58 (95%CI: 1.22-5.45) in the males and 3.78 (95%CI: 1.04;-13.73) in the females]. However, in the multiple adjusted model, the association was only preserved for the males [HR: 2.80 (95%CI: 1.24; 6.31) compared to 2.34 (0.61; 8.96) for the females].^35^

***Non-Alcoholic Fatty Liver Disease in Patients without Metabolic Syndrome and Cardiovascular Risk Factors***

As was mentioned before, overwhelming data suggest that the metabolic syndrome has the strongest relationship with NAFLD.^8^ However, there are no extensive data on the cardiovascular risk in NAFLD patients who do not show the metabolic syndrome. Young patients with NAFLD who do not represent the major components of the metabolic syndrome, including diabetes and hypertension, have the echocardiographic features of early left ventricular dysfunction^37^ and generally show abnormal left ventricular metabolism.^38^ Villanova et al.^39^ reported that the brachial artery endothelial flow-mediated vasodilation was decreased in non-diabetic subjects with NAFLD compared with that in control subjects and this decrease was associated with the histological severity of NAFLD independent of age, sex, insulin resistance, and other variables of the metabolic syndrome. Outstandingly, the 10-year possibility of coronary artery events was moderately increased in these patients.^39^

***Non-Alcoholic Fatty Liver Disease and Left Ventricular Disorders***

NAFLD has also been reportedly associated with functional and anatomical damage to cardiac ventricles. In a study on Indian adolescents, Singh et al.^40^ found that in obese subjects with NAFLD, left ventricular global longitudinal systolic strain and early diastolic strain rates were significantly decreased than lean NAFLD adolescents. Also, the same observation was reported in obese subjects with NAFLD rather than those without NAFLD. In another study on children and adolescents in Turkey, Alp et al.^41^ reported increased end-systolic thickness of the interventricular septum as well as larger left ventricular mass and left ventricular index in NAFLD subjects as compared to controls. They also reported that the interventricular septum was statistically different in the study groups. A study on adult NAFLD patients younger than 55 years of age reported increased thickness of the intraventricular septum and posterior wall as well as larger left ventricular mass/height in the NAFLD group. In the same study, left ventricular systolic function was similar in both groups; however, patients with NAFLD had a lower peak velocity of early and early/late ratio diastolic filing in echocardiography.^37^ In another study on patients with confirmed diagnosis of essential hypertension, NAFLD was diagnosed in 48 (56%) patients and their data were compared to the 38 reaming patients without NAFLD. The patients with NAFLD had similar prevalence of left ventricular hypertrophy compared to the patients without NAFLD but with higher prevalence of diastolic dysfunction, which remained significant even after multivariate analysis.^42^ Another study on patients with essential hypertension, however, showed that NAFLD was associated with insulin resistance but not with increased arterial stiffness.^43^

***Non-Alcoholic Fatty Liver Disease*** ***and*** ***Arterial Hypertension***

A population-based prospective longitudinal study in Germany on about 3200 subjects showed that fatty liver was independently associated with increased diastolic blood pressure and hypertension at baseline and with increased systolic blood pressure and hypertension at follow-up. Individuals with fatty liver had a 3-fold higher chance of hypertension at baseline and follow-up (OR: 2.8, 95%CI: 1.3-6.2 and OR: 3.1, 95%CI: 1.7-5.8, respectively) compared to individuals without fatty liver; all the above-mentioned associations were independent to alcohol consumption.^44^ Another population-based study from Spain showed that NAFLD was independently associated with prevalent hypertension with an adjusted OR of 1.71 (95%CI: 1.10-2.65).^45^ Among non-hypertensive participants, NAFLD was also independently associated with high-normal systolic blood pressure (adjusted OR: 2.13, 95%CI: 1.08-4.20) but not with high-normal diastolic blood pressure. Vasunta et al.,^46^ on the other hand, reported higher day-time and night-time measurements of systolic blood pressure as well as day-time measures of diastolic blood pressure in NAFLD patients than in NAFLD-free subjects.

***Severity of*** ***F*** ***atty*** ***L*** ***iver and*** ***C*** ***ardiovascular*** ***D*** ***iseases***

Severity of NAFLD disease and cardiovascular diseases have also been shown to be associated.^47^ Choi et al.,^48^ in their large study of over 5769 individuals with fatty liver, found that patients with more severe NAFLD were at higher risk for coronary artery disease. In a study in the pediatric context, the authors reported that an increased carotid artery intima-media thickness (IMT), which is a marker of early-generalized atherosclerosis, was associated with higher fatty liver severity.^49^ A significant decrease in the brachial artery flow-mediated vasodilation has also been reported to be allied to the severity of NAFLD histology.^47^ On the other hand, serum levels of adiponectin, as a marker of cardiovascular disease,^50^ have also been reportedly associated with the histological severity of NAFLD.^51^ Similarly, the serum levels of plasminogen activator inhibitor-1 (PAI-1), another proven risk factor for cardiovascular disease, have been associated with the histological severity of NAFLD.^52^ Consequently, according to the current literature, besides its presence, the severity of NAFLD is also a predictor of more severe cardiovascular disease.

***Childhood Fatty Liver***

NAFLD is the most common cause of chronic liver disease in children and adolescents in most of the Western world. In the United States, an autopsy study showed that about 10% of the American population aged 2-19 years had NAFLD, with 38% prevalence among obese ones.^53^ Similar high figures have been reported among children from Asian countries.^54^ The condition in Iran is, albeit better, far from satisfactory.^55^ In a recent retrospective study with a 20-year follow-up period, 66 children with NAFLD underwent a total of about 410 person-years follow-up. In this first report of the long-term prognosis of NAFLD in children, the disease was proven to be able to advance to the end-stage liver disease even in children,^56^ and the log-rank test showed that observed survival free of liver transplantation was significantly shorter in the NAFLD cohort compared to the expected survival in the general United States population of the same age and sex (log-rank test; p value < 0.001). The same study showed that children with NAFLD had a 13.6 (95%CI: 3.8 to 34.8; p value < 0.0001) -fold higher mortality rate or liver transplantation requirement than the general population of the same age and sex. This expands our view on the relevance of the issue in children and alerts us to the significance of the prevention and treatment of this population. In a case-control study of 150 children by Schwimmer et al.,^56^ having NAFLD was associated with the components of the metabolic syndrome, which are strong predictors of cardiovascular morbidity (discussed before). Another study by the same author involving 817 children having undergone autopsy revealed that the prevalence of coronary heart disease was increased by a factor of 2 among those with NAFLD (Schwimmer JB, Deutsch R, Behling C, Lavine JE. Fatty liver as a determinant of atherosclerosis. Hepatology 2005;42:Suppl:610A. abstract). The same study reported that atherosclerosis was significantly more prevalent in children with fatty liver (30% vs. 19%). An interesting observation was a lack of independent association between the body mass index (BMI) and the presence of atherosclerosis, but there was a significant association between fatty liver status and BMI. On the other hand, the odds of having atherosclerosis in obese children were more than 6 times higher among subjects with fatty liver than those without. Nobili et al.,^57^ reporting from the Bogalusa Heart Study in children, demonstrated that the extent of atherosclerotic coverage over the intimal surface was significantly associated with dyslipidemia indices, all of which [total cholesterol/ high-density lipoprotein cholesterol (HDL-c) and low-density lipoprotein cholesterol (LDL-c)/HDL-c] are well-established predictors of cardiovascular diseases.^58^ These data suggest that NAFLD may present as a promoting factor for the cardiovascular threats of overweight and obese children. 

Grade of NAFLD has also been reported as a major factor in cardiovascular risk determination of children with NAFLD. Weghuber et al.^59^ showed that obese children with simple steatosis rather than steatohepatitis seem to have intact vascular function defined by flow-mediated dilation of the brachial artery measured by ultrasound. In an interesting study from Iran, Kelishadi et al.^60^ investigated 100 adolescents and showed that vascular disease in the carotid artery was associated with the ultrasonographic findings of NAFLD. The same findings have been reported in other countries, but because this review article focuses on coronary artery disease and not carotid artery disease, we refer readers to an excellent review article recently published by Pacifico et al.^61^

***Prevention and Treatment***

NAFLD is a disease of a wide-spectrum pathogenesis with reported high levels of mortality.^62^^,^^63^ As a result, the preventive as well as treatment strategies which might have beneficial effects for this disease are also extended, are not applicable to all, and have controversial effectiveness. In our previous studies, we showed that the cardiovascular system is susceptible to insults from seemingly non-associated factors, including hepatic infection by the hepatitis C virus (HCV).^64^^-^^66^ In a study by Sanyal et al.,^67^ investigating differential effects of Pioglitazone, vitamin E, or placebo on non-alcoholic steatohepatitis patients, the authors reported that cardiovascular events occurred with equal frequency in all the three mentioned study groups, although they admitted that their trial might be much too limited to detect meaningful differences in the incidence of cardiovascular events. Several clinical trials have investigated the potential effects of fish oil and/or Omega-3 polyunsaturated fatty acid (n-3 PUFA) consumption on the outcomes of cardiovascular disease. A review on these studies by Wang et al.^68^ showed that fish oil significantly reduced myocardial infarction and cardiac and sudden death, and 33 articles reported the preventive effects of it on primary cardiovascular diseases. Be that as it may, the existing literature also contains controversial reports. Another elaborate review article on the potential effects of fatty fish and n-3 PUFA by Mozaffarian and Wu^69^ showed their very controversial effects on cardiovascular diseases.^69^ In NAFLD patients, Pioglitazone has been proven to have improving effects on the metabolic status.^70^ A recent and very extensive review of literature by Musso et al.^71^ demonstrated that weight loss through lifestyle modifications was safe and improved cardio-metabolic risk profile; statins and polyunsaturated fatty acids improved steatosis; and Thiazolidinediones improved histological disease activity, glucose, lipid, and inflammatory variables and delayed fibrosis progression. Pioglitazone also improved blood pressure. Vitamin E administration was associated with an increase in insulin resistance and plasma triacylglycerols.^71^ Statins are one of the most effective treatment agents in the management of NAFLD.^72^ It also has been suggested that statins are the best drugs in the management of cardiovascular disorders in NAFLD patients.^73^ The same authors in the prospective GREACE study showed that use of statins, especially Atorvastatin, in NAFLD patients was associated with a significantly lower rate of cardiovascular events (10% vs. 30%).^74^ On the other hand, some authors have doubted the effectiveness of statins in reducing cardiovascular risk in NAFLD patients in the absence of dyslipidemia.^75^

Lifestyle interventions such as diet modifications and exercise are fundamental for the treatment of NAFLD. However, like other treatment strategies of NAFLD, they also represent controversial effects on the cardiovascular risk profile. Since a recent review article has assessed this issue thoroughly, we refer readers to this review article by Thoma et al.^76^

## Conclusion

NAFLD is an extremely important disease both in the developed and developing countries due to its high prevalence and high rate of morbidity and mortality. This review article addressed cardiovascular disease in the context of NAFLD because of its prominent role in the adverse effects associated with the disease. The ominous effects of NAFLD are not restricted to its components and even in their absence, NAFLD induces morbidity and mortality rates much higher than those in the general population; and often is associated with the severity of liver disease to the extent that NASH is sometimes considered a cardiovascular-dominated manifestation of NAFLD. Not surprisingly, NAFLD-associated morbidity and mortality even affect children. The treatment strategies for NAFLD and cardiovascular disease are similar and are aimed primarily at lifestyle modifications in order to reduce insulin resistance and other cardiometabolic risk factors. Pharmacotherapy for NAFLD is mostly reserved for patients with more severe disease and patients with high-risk factors for cardiovascular disease. Such patients are candidates for early intervention aimed at preventing cardiovascular diseases and controlling the liver disease itself, bearing in mind that most NAFLD patients will ultimately die from cardiovascular events before advanced liver disease develops.

## Notes

***This paper should be cited as:*** Karbasi-Afshar R, Saburi A, Khedmat H. Heart and Coronary Artery Disease in the Context of Non-Alcoholic Fatty Liver Disease: A Literature Review. J Teh Univ Heart Ctr 2014;9(1):1-8.
